# Integrative transcriptomic analysis reveals diagnostic biomarkers for comorbidity of coronary artery disease and obstructive sleep apnea

**DOI:** 10.3389/fcvm.2025.1658016

**Published:** 2025-09-17

**Authors:** Qingquan Liu, Xiaoyu Chen, Shuhan Chen, Hongzhi Gao, Meiting He, Yingting Shi, Mingzhu Zhao, Liying Yu, Huili Lin

**Affiliations:** ^1^ Cardiovascular Medicine, The Second Affiliated Hospital of Fujian Medical University, Quanzhou, China; ^2^ Department of Endocrinology, The Second Affiliated Hospital of Fujian Medical University, Quanzhou, China; ^3^ Central Laboratory, Fujian Key Laboratory of Lung Stem Cells, The Second Affiliated Hospital of Fujian Medical University, Quanzhou, China; ^4^ Department of Bioinformatics, School of Medical Technology and Engineering, Fujian Medical University, Fuzhou, Fujian, China

**Keywords:** coronary artery disease, obstructive sleep apnea syndrome, RNA sequencing, biomarker, immune

## Abstract

**Background:**

The co-occurrence of coronary artery disease (CAD) and obstructive sleep apnea (OSA), termed CADOSA, leads to worse clinical outcomes than either condition alone, yet its molecular mechanisms remain unclear, necessitating biomarker discovery for improved diagnosis and personalized management.

**Methods:**

This study enrolled 96 age-matched participants (24 healthy controls, 25 CAD, 23 OSA, and 24 CADOSA) for clinical assessment and PBMC transcriptomic profiling. Integrated bioinformatics analyses included differential gene expression (edgeR/DESeq2), pathway enrichment, protein-protein interaction networks and topological analysis, machine learning-based biomarker selection, and immune cell infiltration evaluation.

**Results:**

CADOSA patients had more severe cardiac dysfunction (enlarged left ventricle), respiratory impairment (higher apnea-hypopnea index), and metabolic disturbances (elevated triglycerides/creatinine) compared to single-disease condition of CAD or OSA. Transcriptomics identified 832 CAD-specific, 166 OSA-specific, and 376 CADOSA-specific DEGs compared to the healthy control. The CADOSA exhibited both shared (impaired efferocytosis, neutrophil extracellular traps, and cytoskeletal abnormalities) and unique enriched pathways (NOD-like receptor/PPAR signaling pathway), predominantly associated with immune or metabolic dysregulation. Enhanced expression of S100A12 and MMP9 genes (AUC = 0.83 and 0.78, respectively) was identified as potential biomarkers for CADOSA, and their upregulation was further confirmed by qRT-PCR. Notably, S100A12 expression was correlated with increased monocyte infiltration, highlighting its role in inflammatory pathogenesis of CADOSA.

**Conclusions:**

These findings reveal immune-metabolic dysregulation underlying CADOSA and provide potential diagnostic biomarkers and targeted therapeutic targets (S100A12 and MMP9) for CADOSA patients.

## 1 Introduction

Coronary artery disease (CAD) and obstructive sleep apnea syndrome (OSA) are two widespread diseases that present considerable worldwide health challenges. CAD is among the most prevalent and severe cardiovascular diseases globally, remaining the leading cause of mortality worldwide. It accounts for approximately 17.9 million deaths annually, primarily due to atherosclerosis-driven complications such as myocardial infarction and heart failure (1, 2). Simultaneously, OSA is marked by recurrent episodes of upper airway obstruction during sleep. It affects nearly one billion adults worldwide and shows a notably elevated prevalence in individuals with obesity, hypertension, and cardiovascular comorbidities (3). The coexistence of CAD and OSA is particularly alarming. Studies report that 40%–60% of CAD patients also have OSA, which exacerbates cardiovascular risks such as acute coronary syndromes, arrhythmias, and sudden cardiac death (4–6). Despite advances in treatment strategies, clinical outcomes remain be suboptimal in patients with both CAD and OSA. This underscores the necessity to clarify the shared pathological mechanisms that may contribute to comorbid disease (7). Identifying biomarkers for CADOSA comorbidity is therefore crucial. Such biomarkers would support the development of precise diagnostic tools and facilitate targeted therapeutic interventions for this high-risk population.

The pathogenesis of both disease conditions converges on chronic inflammation, endothelial dysfunction, and oxidative stress (8). In CAD, principal mediators include pro-inflammatory cytokines (e.g., IL-6, TNF-α) (9), adhesion molecules (e.g., ICAM-1, VCAM-1) (10), and aberrant lipid metabolism (e.g., APOE, LDLR) (11). Likewise, the pathophysiology of OSA encompasses intermittent hypoxia-induced HIF-1αsignaling, which sustains systemic inflammation mediated by NF-κB, and oxidative damage (12). Interconnected mechanisms, including endothelial impairment, immune activation, and oxidative stress, suggest possible molecular connections between CAD and OSA (13). OSA-related intermittent hypoxia exacerbates endothelial dysfunction, hence accelerating atherosclerotic development in CAD (14). However, the specific molecular pathways that mediate comorbid CAD and OSA are little characterized, especially in peripheral immune cells that function as systemic indicators of vascular inflammation.

Transcriptomic profiling, particularly RNA sequencing (RNA-seq), has become as an effective method for elucidating the molecular foundations of complex diseases. Transcriptomic analyses have revealed the molecular processes of CAD and OSA independently. In CAD, transcriptomic studies have identified differentially expressed genes (DEGs) associated with inflammation, lipid metabolism, and vascular remodeling (15–17). In OSA, alterations in genes are involved in hypoxia response, oxidative stress, and immune regulation (18, 19). Nevertheless, researches on CAD and OSA comorbidity are limited, especially with peripheral blood mononuclear cells (PBMCs) in comorbid conditions, which provide a non-invasive perspective on systemic immunological activation. The functional significance of common transcriptomic changes biomarkers or therapeutic targets remains largely unexplored.

In this study, we performed extensive RNA-seq of PBMCs from four cohorts: healthy controls, CAD-only (CAD), OSA-only (OSA), and CADOSA (CADOSA) patients. Through the integration of differential expression analysis, functional enrichment, protein-protein interaction, machine learning, and immune cell infiltration analyses, we aimed to identify critical molecular pathways and molecules associated with the OSA, CAD and their comorbidity. Our findings will provide novel insights into the common and unique molecular pathways of CAD and OSA, facilitating the development of diagnostic and targeted therapeutic strategies.

## 2 Materials and methods

### 2.1 Description of the study cohort and sample collection

The study cohort comprised participants diagnosed with OSA and/or CAD, and healthy controls. We consecutively screened and recruited patients admitted for coronary angiography and polysomnography (PSG) based on clinical indications. Participants diagnosed with CAD were selected based on evaluations by two cardiovascular specialists through CT coronary angiography or conventional coronary angiography. Exclusions included for those with severe infectious diseases, other cardiac conditions inducing angina (such as hypertrophic cardiomyopathy, myocarditis, or severe valvular heart disease), active malignancies or chemotherapy treatment, severe cardiac dysfunction, pacemaker implantation, frequent ectopic beats exceeding >10% of total heartbeats, prior revascularization procedures (coronary angioplasty or bypass grafting), major surgeries conducted within the past six months, or prior continuous positive airway pressure therapy. Patients with OSA were included if diagnosed via polysomnography (PSG) with an apnea-hypopnea index (AHI) ≥5. While excluding those who had been hospitalized for heart failure or respiratory disease in the past six weeks, were unable to undergo PSG, required oxygen therapy, had airflow-limiting conditions, or presented with renal insufficiency or long-term medication usage. Healthy volunteers without relevant diseases were recruited as controls. All subjects granted written informed consent, and the study was approved by the Institutional Review Board of the Second Affiliated Hospital of Fujian Medical University [NO. 597(2023), Quanzhou, Fujian, China]. Totally, 23 OSA, 25 CAD, and 24 CADOSA patients were enrolled, with health controls (*n* = 24) included for comparative analysis.

Clinical characteristics, including age, gender, body mass index (BMI), and relevant medical history, were documented as comprehensively as possible for each participant (Supplementary Table S1). Blood samples were taken and followed by PBMCs isolation using Ficoll-Paque density gradient centrifugation within two hours. The PBMCs were washed twice with phosphate-buffered saline and resuspended in Trizol reagent for RNA extraction, and then kept at −80℃ for RNA sequencing.

### 2.2 Preprocessing of RNA sequencing data

Total RNA was extracted utilizing the TRIzol Reagent (ThermoScientific, cat. #15596026) following the manufacturer's protocol. Subsequently, genomic DNA was removed by subjecting the samples to DNase I to eliminate DNA contamination. RNA quality and quantity were evaluated using the Agilent 2,100 Bioanalyzer and the NanoDrop™ OneC spectrophotometer, respectively. Samples with an RNA Integrity Number (RIN) >7.0 and a 260/280 ratio between 1.8 and 2.1 were preserved for subsequent analysis. RNA sequencing libraries were constructed and sequenced on the DNBSEQ-T7 platform (MGI), producing 150 bp paired-end reads. The raw sequencing data were processed through the following steps: (1) Adapter sequences and low-quality bases were trimmed using fastp (v0.23.2) with the following parameters: –dont_eval_duplication –qualified_quality_phred 20 –unqualified_percent_limit 8. (2) Clean reads were aligned to the human reference genome (GRCh38) using STAR (v2.7.6a, parameters: outFilterMatchNminOverLread 0.66 –outSAMmapqUnique 60 –chimOutJunctionFormat 1 –chimSegmentMin 10 –chimOutType SeparateSAMol). (3) Gene-level counts were quantified utilizing featureCounts (v1.5.1) with parameters of -d 30 -D 1,000 -C -s 0 -t exon -g fcid –primary -O.

### 2.3 Identification of differentially expressed genes (DEGs)

Principal component analysis (PCA) was performed on Fragments Per Kilobase of exon model per Million mapped fragments (FPKM)- normalized gene expression data to reduce dimensionality and visualize sample clustering patterns based on overall transcriptomic variation. DEGs between each disease (OSA, CAD, CADOSA) group and the control group based on raw count data were identified using the edgeR and DESeq2 R package (v3.40.2). Initial quality filtering excluded genes detected in fewer than 25% of samples. Genes with an adjusted *P*-value (Benjamini-Hochberg correction) <0.05 and an absolute fold change >1.5 were considered significant. To ensure robust results, only genes consistently identified by both edgeR and DESeq2 analytical methods were retained as disease-associated DEGs. The Kyoto encyclopedia of genes and genomes (KEGG) enrichment of DEGs were performed utilizing the ClusterProfiler R package. The KEGG pathways with *P* < 0.05 were considered significantly enriched. The top 20 or significantly (*P* < 0.05) enriched functional pathways were visualized. Comparative analysis of DEGs from CADvsHC, OSAvsHC, and CADOSAvsHC through Venn diagramming enabled identification of condition-specific DEGs (unique to CAD or OSA) and comorbidity-associated DEGs (shared across all comparisons).

### 2.4 Gene expression clustering and core gene selection

The Mfuzz (v2.58.0) R package was used to examine temporal gene expression patterns across disease progression during “HC→CAD/OSA→CADOSA” development based on FPKM- normalized gene expression values. The cluster number of Mfuzz were set as 6. Genes with statistically significant clusters (*P* < 0.05) retained for downstream analysis. Core genes for CAD and OSA were defined as the intersection between disease-specific DEGs and genes exhibiting inverted V-shaped expression patterns that peaked in the respective disease state. For CADOSA comorbidity, core genes were identified as those demonstrating both upward-trending expression patterns (maximal in CADOSA) and significant overlap with key comorbidity-associated DEGs.

### 2.5 Protein network analysis and hub gene identification

To elucidate key molecular interactions, we established protein-protein interaction (PPI) networks for disease-specific DEGs or CADOSA-core DEGs using the STRING database. The resulting interaction data were imported into Cytoscape for topological analysis. Gene centrality was systematically evaluated through five distinct algorithms implemented in the CytoHubba plugin: Maximal Clique Centrality (MCC), Edge Percolated Component (EPC), Closeness, Degree, and Betweenness. Hub genes were rigorously defined as those consistently ranked among the top 10 most central nodes across all five-network metrics, ensuring the identification of biologically relevant and highly connected molecular regulators.

### 2.6 Machine learning-based biomarkers selection

To identify optimal diagnostic biomarkers, we implemented a comprehensive machine learning pipeline. First, candidate genes were obtained from the intersection of core and hub genes. To develop predictive model of each disease, transcriptomic data was individually and randomly divided into training (70%) and validation (30%) sets. We then applied three distinct machine learning algorithms to prioritize robust biomarkers: (1) Elastic Net regression (with optimal regularization parameter *λ* = 0.05), (2) Random Forest, and (3) Support Vector Machine (SVM). Machine learning models were built using 10-fold cross-validation to improve generalizability. Final biomarker selection was determined by consensus between genes with non-zero coefficients form Elastic Net (indicating strong predictive contribution) and top-ranked genes based on variable importance (Mean Decrease Gini) from Random Forest. The performance of combined diagnostic model using all candidate and individual biomarker was systematically evaluated using ROC analysis.

### 2.7 Estimation of immune infiltration

The abundances of immune cells in four group samples were assessed individually using CIBERSORT algorithms. The difference in cell proportion was statistically evaluated. The associations between cells and biomarker genes were calculated and visualized using “ggcor” R package.

### 2.8 Quantitative PCR for gene quantification

Gene expression of CADOSA biomarkers were verified using cDNA samples from 12 CADOSA and 12 HC cohorts. Quantitative PCR was performed in 10 μl reactions containing TB Green Premix Ex Taq II, gene-specific primers (MMP9, S100A12), and cDNA template, using the following cycling conditions: 95 °C for 30 s, then 40 cycles of 95°C for 5 s and 60 °C for 30 s Gene expression was calculated by the 2^(-ΔΔCt) method using β-actin as the endogenous control. The primers were synthesized by Sangon Biotech (Shanghai, China), and their sequences are: S100A12-forward primer 5′-GGGGCATTTTGACACCCTCT-3′ and S100A12-reverse primer 5′-CGCAATGGCTACCAGGGATA-3′; GLUD2-forward primer 5′-CAGCATCGTGGAGGACAAGT-3′ and GLUD2-reverse primer 5′-CGGTAGCCTTCGATGACCTC-3′; β-actin-forward primer 5′-GAGAAAATCTGGCACCACACC-3′, β-actin-reverse primer 5′-GGATAGCACAGCCTGGATAGCAA-3′.

### 2.9 Statistical analysis of clinical factors

Statistical analyses of clinical variables were performed to compare the demographic and clinical characteristics among the control, OSA, CAD, and CADOSA groups. All statistical analyses in this study were conducted using SPSS 27.0 software. The normality and homogeneity of variance for all factors were evaluated using the Kolmogorov–Smirnov test. Categorical variables, such as gender, were presented as frequencies (%) and analyzed using the Chi-square test. Continuous variables that follow a normal distribution are represented as mean ± standard deviation (SD), while non-normally distributed continuous variables are shown as median (interquartile range, IQR). One-way ANOVA was employed for intergroup comparisons of normally distributed continuous variables with homogeneous variance. Upon detecting significant differences were detected (*P* < 0.05), *post-hoc* pairwise comparisons were conducted using either LSD (for homogeneous variance) or Tamhane's T2 method (for heterogeneous variance). For data that is not normally distribute or exhibits non-homogeneous variance, the Kruskal–Wallis *H*-test was employed for intergroup comparisons, followed by Bonferroni-adjusted pairwise comparisons upon the identification of significant differences. All tests were two-tailed, with a *P* < 0.05 was considered statistically significant.

## 3 Results

### 3.1 Cohort characteristics

We compared demographic and clinical variables across four cohort groups (Table 1): healthy controls (HC, *n* = 24), CAD (*n* = 25), OSA (*n* = 23), and comorbid CADOSA (*n* = 24). No significant differences were observed in age, BMI, lipid profiles, and liver enzymes among the four groups. Marked disparities in gender distribution were noted (*P* = 0.002), with a higher male predominance in the OSA and CADOSA cohorts (76% and 91.7%, respectively). Statistical comparisons revealed that the heart rate in the OSA group was significantly higher than in the HC group (*P* < 0.05), which may be attributed to chronic nocturnal hypoxia-induced sympathetic overactivation. Regarding blood biochemical indices, the CADOSA group exhibited significantly higher levels of total triglycerides (TG) and creatinine (CR) compared to the CAD group (*P* < 0.05), suggesting that comorbid CAD and OSA exacerbates TG metabolic dysregulation and renal impairment. Among myocardial injury markers, the CAD group demonstrated significantly elevated D-dimer, NT-proBNP, and Hs-TnT levels compared to either the OSA or HC groups (*P* < 0.05), confirming increased thrombotic risk and myocardial damage in CAD patients. Echocardiographic parameters showed progressive increases in left ventricular end-diastolic diameter (LVD) and left ventricular end-systolic diameter (LVS) from HC→OSA→CAD→CADOSA, with the CADOSA group exhibiting significantly higher values than HC and/or OSA groups (*P* < 0.05), indicating that comorbid OSA aggravates cardiac systolic dysfunction in CAD patients. Polysomnography results demonstrated significantly higher AHI values and lower SpO2 levels in both OSA and CADOSA groups compared to HC and CAD groups (*P* < 0.05), reflecting substantially impaired respiratory function in OSA and comorbid CADOSA patients. Furthermore, we observed a progressive, though statistically non-significant, increase in the prevalence of carotid plaque, left ventricular hypertrophy, and valvular regurgitation across the spectrum from HC→OSA→CAD→CADOSA. This trend suggests that the synergistic effects of CAD-induced myocardial ischemia and OSA-mediated intermittent hypoxia may potentiate the development of these pathological manifestations, potentially exacerbating disease severity of CAD and OSA.

**Table 1:** Demographic and clinical characteristics of the participants at baseline.

Characteristic	HC (*n* = 24)	OSA (*n* = 23)	CAD (*n* = 25)	CADOSA (*n* = 24)	*P* value	Comparison^#^
Demographic characteristics						
Age, years	57 (48.0,70.0)	59 (48.0,70.0)	69 (60.0,74.0)	64.5 (55,71.75)	0.116	
Gender*					0.002	
Male, *n* (%)	10 (41.7%)	16 (69.6%)	19 (76.0%)	22 (91.7%)		
Female, *n* (%)	14 (58.3%)	7 (30.4%)	6 (24.0%)	2 (8.3%)		
BMI, kg/m^2^	24.27 ± 3.48	25.65 ± 4.53	23.17 ± 4.08	25.57 ± 3.68	0.11	
Underweight (<18.5), *n* (%)	1 (4.1%)	2 (8.7%)	3 (12.0%)	1 (4.1%)		
Normal weight (18.5–23.9), *n* (%)	7 (29.2%)	6 (26.1%)	11 (44.0%)	6 (25.0%)		
Overweight (24–27.9), *n* (%)	7 (29.2%)	8 (34.8%)	7 (28.0%)	10 (41.7%)		
Obesity (>28), *n* (%)	4 (16.7%)	7 (30.4%)	3 (12.0%)	7 (29.2%)		
No data, *n* (%)	5 (20.8%)	/	1 (4.0%)	/		
HR*, beats/min	75.55 ± 5.60	83.61 ± 11.61	77.24 ± 16.39	80.42 ± 12.41	0.029	1 < 2
Blood Pressure						
SBP, mmHg	125.5 (120.8,148.8)	133 (124.0,150.0)	128 (121.0,141.0)	131 (123.0,140.0)	0.672	
DBP, mmHg	78.20 ± 7.82	86.78 ± 14.85	81.96 ± 11.26	80.25 ± 10.14	0.122	
Blood routine test						
NEU, 10^−9^/L	3.80 (3.00,4.52)	4.20 (3.33,5.66)	4.50 (3.36,6.42)	4.32 (3.18,4.91)	0.221	
RBC,10^−12^/L	4.69 ± 0.58	4.75 ± 0.58	4.50 ± 0.52	4.40 ± 0.72	0.173	
HGB, g/L	135.46 ± 19.12	145.39 ± 17.99	138.32 ± 14.90	135.46 ± 22.63	0.235	
Blood biochemistry						
AST, U/L	23.6 (19.9,33.95)	23.1 (18.7,30.0)	20.7 (17.8,50.85)	22.2 (17.18,29.28)	0.967	
ALT, U/L	20.8 (13.58,40.45)	22.3 (14.4,31.8)	19.7 (13.05,36.3)	21.3 (14.88,37.9)	0.988	
TC, mmol/L	4.84 ± 0.91	4.47 ± 0.32	4.58 ± 1.07	4.57 ± 1.21	0.634	
TG*, mmol/L	1.81 (1.18,2.43)	1.7 (0.9,2.01)	1.11 (0.89,1.8)	1.88 (1.38,2.71)	0.024	3 < 4
HDL, mmol/L	1.25 ± 0.38	1.19 ± 0.32	1.23 ± 0.37	1.04 ± 0.20	0.120	
LDL, mmol/L	3.08 ± 0.89	2.79 ± 0.86	2.90 ± 0.98	2.89 ± 1.17	0.789	
CR*, umol/L	63.5 (51.5,73.5)	70 (61.0,90.0)	69 (61.5,74.5)	75.5 (63.75,101)	0.043	1 < 4
UA, mmol/L	330.71 ± 82.92	364.91 ± 108.56	348.72 ± 99.42	368.71 ± 83.36	0.490	
Glucose, mmol/L	5.06 (4.84,5.91)	5.38 (5.06,6.2)	5.64 (5.19,7.04)	5.57 (5.05,6.29)	0.149	
HbA1C, %	5.7 (5.4,6.7)	5.6 (5.48,6.7)	6.15 (5.68,7.1)	6.2 (5.75,6.8)	0.289	
Indicators of myocardial injury						
CK-MB, U/L	12.15 (11.1,14.93)	13.8 (11.1,15.5)	15.4 (12.25,23.25)	14.05 (12.23,16.58)	0.103	
D-dimer*, ug/ml	0.29 (0.17,0.46)	0.2 (0.12,0.33)	0.55 (0.27,1.61)	0.29 (0.19,1.08)	0.007	2 < 3
NT-proBNP*, pg/ml	62.79 (24.23,335.05)	57.38 (26.98,131.28)	263.98 (104.8,1013.2)	115.67 (31.46,311.2)	0.01	2 < 3
Hs-TnT*, ng/L	7.89 (5.3,12.27)	9.0 (5.65,12.11)	12.75 (9.23,106.1)	9.31 (7.24,19.75)	0.026	1 < 3
Carotid artery ultrasound						
Carotid plaque	0.237					
Yes, *n* (%)	3 (12.5%)	3 (13.0%)	5 (20.0%)	8 (33.3%)		
No, *n* (%)	21 (87.5%)	20 (87.0%)	20 (80.0%)	16 (66.7%)		
Echocardiography						
LVEF, %	64.5 (61.0,68.0)	67 (64.0,70.0)	66.5 (56.5,68)	66 (59.0,68.0)	0.154	
LVD*, mm	45.56 ± 3.38	46.43 ± 4.50	48.82 ± 5.99	49.43 ± 3.69	0.019	1 < 3, 4; 2 < 4
LVS*, mm	29 (28,31)	29 (27,32)	30 (28.75,37)	32 (30,35)	0.008	1, 2 < 4
IVS, mm	10.61 ± 2.28	10.83 ± 1.85	11.00 ± 1.69	11.17 ± 1.34	0.776	
LVPW, mm	10 (9,10.25)	10 (9,11)	10 (9,11)	10 (10,12)	0.244	
Valvular regurgitation					0.261	
Yes, *n* (%)	14 (58.3%)	16 (69.6%)	19 (76.0%)	20 (83.3%)		
No, *n* (%)	10 (41.7%)	7 (30.4%)	6 (24.0%)	4 (16.7%)		
Left ventricular hypertrophy				0.143		
Yes, *n* (%)	3 (12.5%)	7 (30.4%)	6 (24.0%)	10 (41.7%)		
No, *n* (%)	21 (87.5%)	16 (69.6%)	19 (76.0%)	14 (58.3%)		
Sleep evaluation						
AHI*, events/h	2.2 (1.15,3.95)	15.2 (11.8,25.5)	3.5 (1.0,4.3)	18.1 (9.2,31.9)	<0.001	1 < 2, 4; 3 < 2, 4
SpO_2_*, %	89 (88.5,90.5)	83 (80,85)	89 (88,92)	84 (81,88)	<0.001	2 < 1, 3; 4 < 3

* Marks a significant difference in the clinical factor among the HC, OSA, CAD, and CADOSA groups (*P* value < 0.05).

^#^ LSD (Least Significant Difference) method or T2 method or Bonferroni method was used for pairwise comparison within groups, 1 = HC, 2 = OSA, 3 = CAD, 4 = CADOSA.

Values are mean ± SD, *n* (%), or median (interquartile range), unless otherwise indicated. Differences in clinical and imaging parameters between groups were compared using one-way ANOVA.

N, number of patients; BMI, body mass index; HR, heart rate; SBP, systolic blood pressure; DBP, diastolic blood pressure; NEU, neutrophils; RBC, red blood cells; HGB, hemoglobin; TBIL, total bilirubin; AST, aspartate aminotransferase; ALT, alanine aminotransferase; TC, total cholesterol; TG, triglycerides; HDL, high density lipoprotein; LDL, low density lipoprotein; CR, creatinine; UA, uric acid; HbA1C, glycated hemoglobin A1C; CK-MB, creatine kinase, MB Form; NT-proBNP, N-terminal pro-B-type natriuretic peptide; LVEF, left ventricular ejection fraction; LVD, left ventricular end-diastolic diameter; LVS, left ventricular end-systolic diameter; IVS, interventricular septal thickness; LVPW, left ventricular posterior wall thickness; AHI, apnea hypopnea index; SpO_2_, peripheral oxygen saturation.

### 3.2 Transcriptomic profiles of CAD

**Figure 1:**
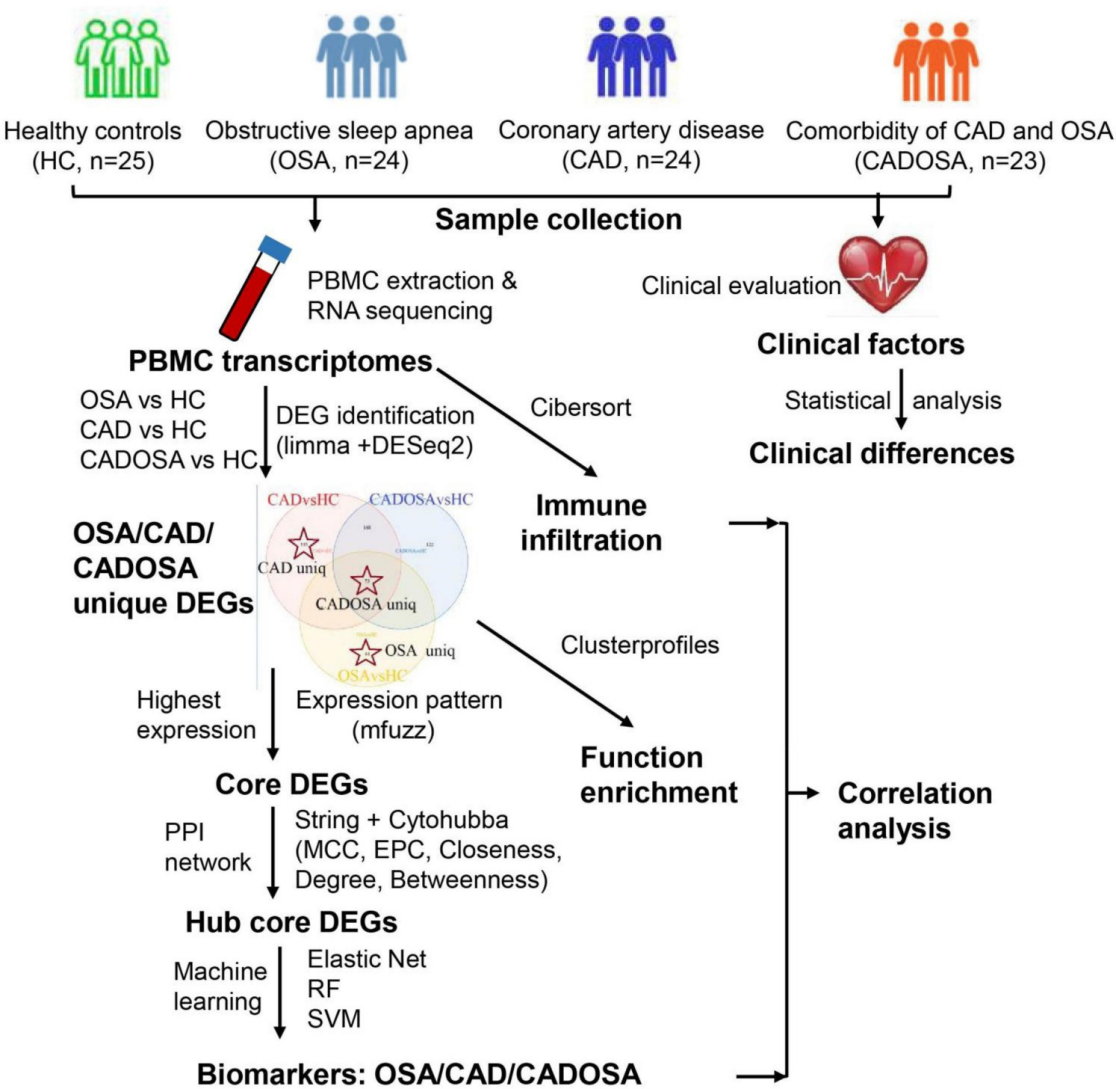
Workflow of this study.

The RNA-seq yielded sequencing data size ranging from 5.44 Gbp to 7.05 Gbp for each sample across four groups, resulting in 673.74 Gbp data in total (Supplementary Table S2). After quality filtration, >90% of reads were preserved as clean data for subsequent reads mapping, gene quantification and differential expression analysis. The detailed workflow of this study was shown as Figure 1.

**Figure 2:**
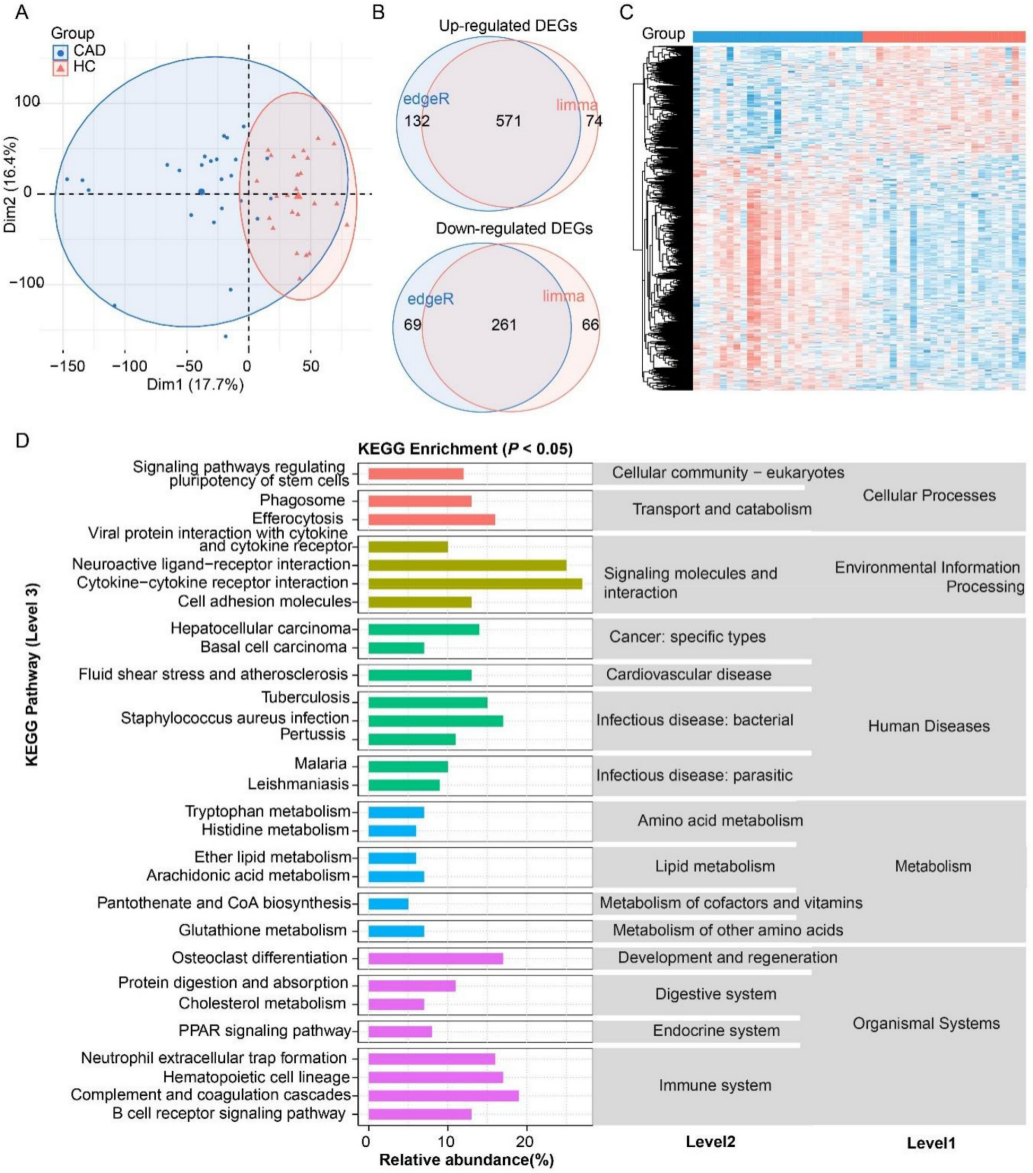
Analysis of differentially expressed genes (DEGs) in coronary artery disease (CAD) compared to the healthy control (HC). **(A)** Principal component analysis (PCA) confirming distinct transcriptomic profiles between the CAD and HC. **(B)** Venn diagram of DEG number identified by edgeR and DESeq2 methods. **(C)** Heatmap of DEGs. **(D)** KEGG pathway enrichment of DEGs (*P* < 0.05).

Principal component analysis based on transcriptomic gene expression showed that CAD and HC samples were apparently distinguished, indicating an intrinsic difference in gene regulation between the two groups (Figure 2A). The limma and DESeq2 methods were combined to identify commonly and DEGs in the CAD. As a result, the CAD group exhibited 571 up-regulated and 261 down-regulated DEGs (Supplementary Table S3), with over two-thirds being up-regulated and nearly one-third being down-regulated (Figures 2B,C). These 832 DEGs were significantly enriched in 30 KEGG pathways (level 3 in Figure 2D), which were classified into 15 level 2 categories and five level 1 pathways. Notably, “immune system” and “signaling molecules and interaction” were the top two abundantly enriched categories. The immune-related pathways, including “Cytokine–cytokine receptor interaction”, “B cell receptor signaling pathway,” “Complement and coagulation cascades”, and “Neutrophil extracellular trap formation”, exhibited prominent enrichment, suggesting a robust involvement of immune regulation in the CAD transcriptomic changes. The signaling molecules and interaction pathways such as “Neuroactive ligand–receptor interaction”, “cytokine-cytokine receptor interaction”, and “cell adhesion molecules” were significantly enriched, highlighting the critical role of intercellular communication in CAD development. Metabolic reprogramming was another key feature of the CAD, with DEGs strongly associated with “Tryptophan metabolism”, “Histidine metabolism”, “Glutathione metabolism”, “Ether lipid metabolism”, and “Arachidonic acid metabolism”. These findings underscore the importance of amino acid and lipid metabolism in the biological regulation of the CAD. Furthermore, pathways related to “phagosome” and “efferocytosis” were enriched, indicating heightened immune clearance activity and potential involvement in inflammation, tissue homeostasis. In the context of human diseases, DEGs were enriched in “Hepatocellular carcinoma”, “Basal cell carcinoma”, “Tuberculosis”, and “Malaria”, suggesting a possible link between the identified genes and infection or oncogenic processes. Collectively, these results demonstrate that the DEGs are not only pivotal in immune and signaling pathways but also play a central role in metabolic adaptation, particularly in amino acid and lipid metabolism, which may have broader implications for the CAD mechanisms and therapeutic targeting.

### 3.3 Transcriptomic shifts in OSA

**Figure 3:**
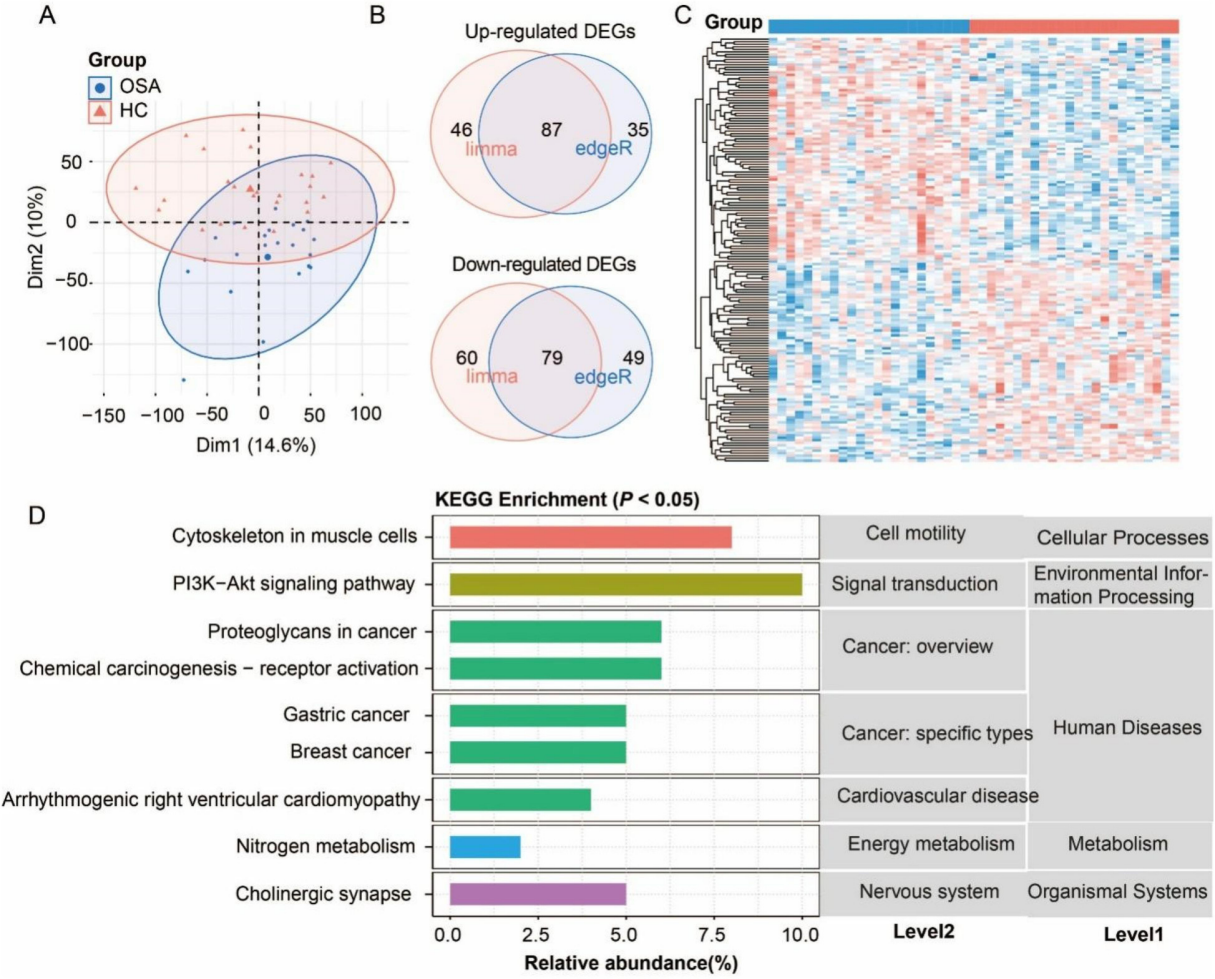
Analysis of differentially expressed genes (DEGs) in obstructive sleep apnea (OSA) compared to the healthy control (HC). **(A)** Principal component analysis (PCA) confirming distinct transcriptomic profiles between the OSA and HC. **(B)** Venn diagram of DEG number identified by edgeR and DESeq2 methods. **(C)** Heatmap of DEGs. **(D)** KEGG pathway enrichment of DEGs (*P* < 0.05).

Samples from the OSA and HC groups exhibited clear separation by PCA analysis, confirming distinct transcriptomic profiles (Figure 3A), with limma and DESeq2 identifying 87 up-regulated and 79 down-regulated DEGs (Figures 3B,C; Supplementary Table S4). KEGG enrichment analysis revealed these DEGs were significantly associated with cytoskeleton in muscle cells (level 3) of cell motility (level 2), PI3K-Akt signaling of signal transduction, arrhythmogenic right ventricular cardiomyopathy of cardiovascular disease, nitrogen metabolism of energy metabolism, and cholinergic synapse of nervous system (Figure 2D). While cancer-related pathways like proteoglycans in cancer were enriched, their relevance likely relates to shared oxidative stress mechanisms rather than direct oncogenesis. These multi-systemic pathway alterations collectively demonstrate how chronic intermittent hypoxia in OSA drives molecular changes affecting cardiovascular integrity, neuromuscular function, and metabolic homeostasis, providing a framework for understanding OSA-related complications.

### 3.4 Gene regulation in CADOSA comorbidity

**Figure 4:**
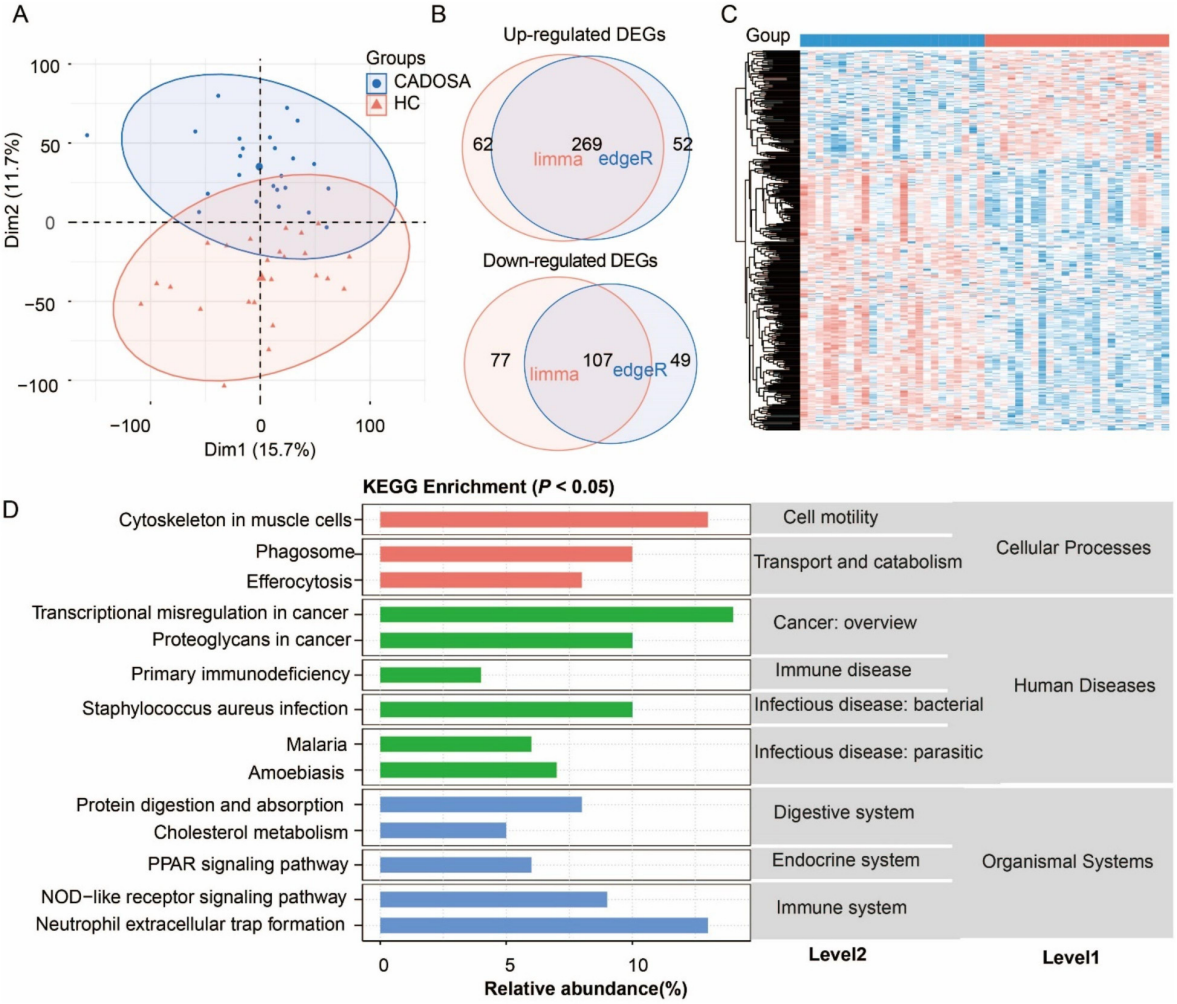
Analysis of differentially expressed genes (DEGs) in coronary artery disease (CAD) complicated with OSA (CADOSA) compared to the healthy control. **(A)** Principal component analysis (PCA) confirming distinct transcriptomic profiles between the CADOSA and HC. **(B)** Venn diagram of DEG number identified by edgeR and DESeq2 methods. **(C)** Heatmap of DEGs. **(D)** KEGG pathway enrichment of DEGs (*P* < 0.05).

Similarly, the CADOSA samples were clustered and separated from HC samples (Figure 4A). Compared to healthy controls, CADOSA exhibited 269 up-regulated and 107 down-regulated DEGs (Figures 4B,C, Supplementary Table S5), showing more DEGs than OSA alone but fewer than CAD alone. KEGG enrichment analysis revealed these DEGs were significantly associated with human diseases, organismal systems and cellular processes. The dysregulation of immune-related pathways included “NOD-like receptor signaling pathway”, and “neutrophil extracellular trap formation” suggests chronic inflammation. Other consisted of the “PPAR signaling pathway” in endocrine system and “protein digestion and absorption” in digestive system might indicate body function disturbances. Similar to the CAD, the comorbidity also exhibited the same enriched pathways of cell motility (cytoskeleton in muscle cells), and transport and catabolism (phagosome and efferocytosis). The infection-related pathways (such as “Staphylococcus aureus infection” and “malaria”) and primary immunodeficiency in human diseases reflects immune vulnerability. These multi-systemic alterations highlight the synergistic effects of CAD and OSA, where chronic intermittent hypoxia might exacerbate both immune dysregulation and cardiovascular damage.

### 3.5 Comparative analysis of KEGG pathways and hub genes relevels divergent molecular regulations among OSA, CAD, and their comorbidity

**Figure 5:**
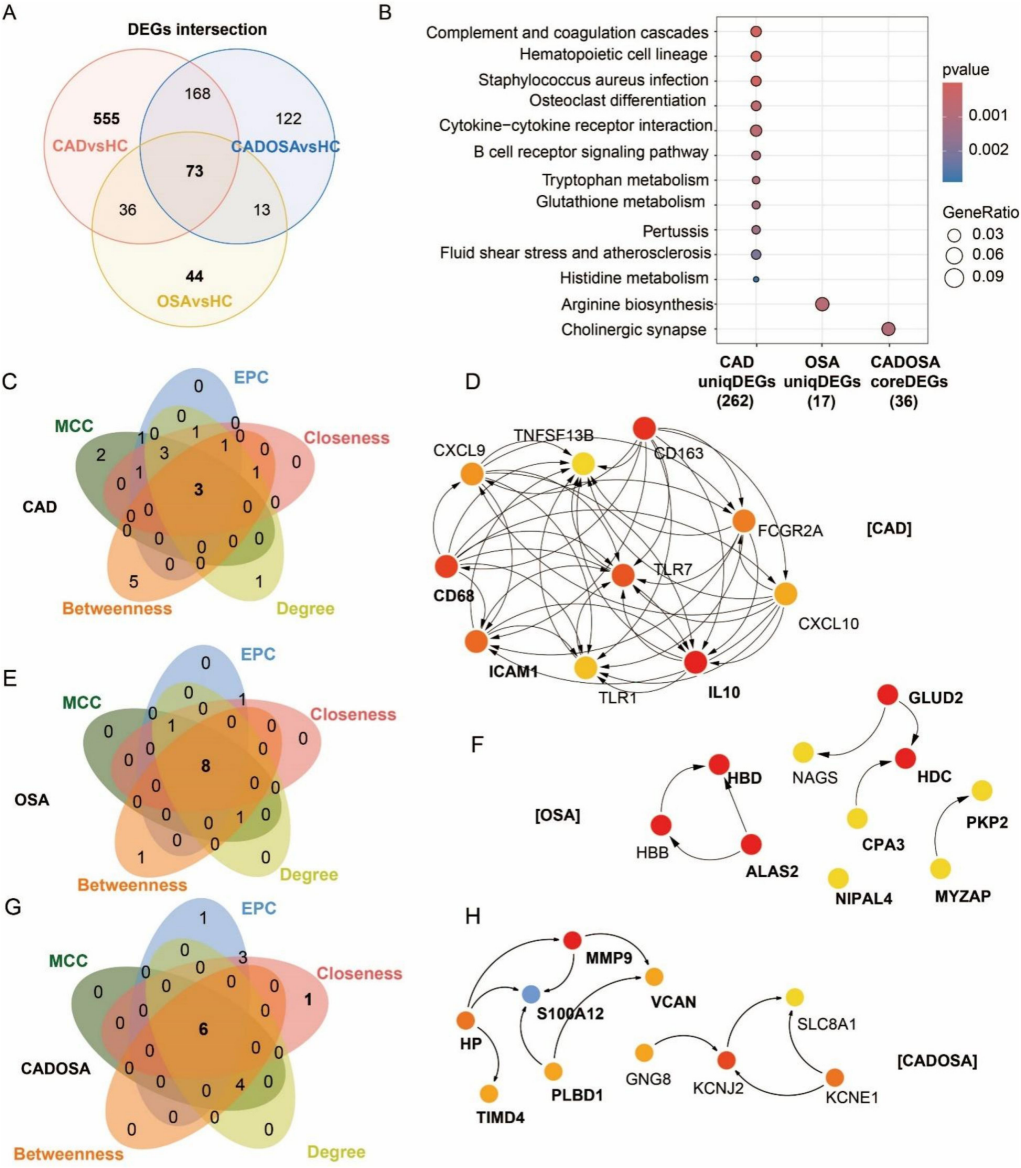
Differentially expressed genes (DEGs) and functional enrichment comparisons and hub genes in CAD, OSA, and their comorbidity. **(A)** Venn of DEGs number across three comparisons: CAD vs. healthy controls (HC), OSA vs. HC, and CADOSA vs. HC. **(B)** Comparisons of KEGG enrichment analysis of CAD-specific and OSA-specific DEGs, and key DEGs shared in CADOSA comorbidity. **(C–H)** Venn diagram and Protein-protein interaction (PPI) MCC-network of the top 10 hub genes of CAD, OSA, and CADOSA identified by five network centrality algorithms: Maximal Clique Centrality (MCC), Edge Percolated Component (EPC), Closeness, Degree, and Betweenness. Overlapping genes represent consensus hub candidates. (Node color intensity reflects centrality ranking (dark red = highest, light yellow = lowest). Bold labels indicate genes consistently prioritized by all five algorithms.

To investigate the distinct molecular profiles among CAD, OSA, and CADOSA, we identified unique and shared DEGs across the three comparisons. The Venn diagram revealed 555 CAD-specific DEGs and 44 OSA-specific DEGs, and 73 CADOSA-core DEGs which were commonly shared in three comparisons (CAD vs. HC, OSA vs. HC, and CADOSA vs. HC) (Figure 5A). Pathway enrichment revealed distinct profiles of these disease-specific and comorbidity-core DEGs, with CAD exhibited the most complex molecular regulations (Figure 5B). The CAD-specific DEGs were predominantly associated with immune dysregulation (complement and coagulation cascades, hematopoietic cell lineage, and B cell receptor signaling pathway) and metabolic disturbances (glutathione/tryptophan/histidine metabolism). The OSA-specific DEGs showed enrichment in arginine biosynthesis, while the shared DEGs in CADOSA converged on cholinergic synapse.

To further elucidate the molecular interactions, we constructed protein-protein interaction (PPI) networks using STRING and identified hub genes for each disease group. Hub gene selection was performed using five topological algorithms (MCC, EPC, Closeness, Degree, and Betweenness) implemented in the CytoHubba plugin for Cytoscape, and defined as with the intersection of top-ranked 10 genes across all five methods. Analysis results revealed distinct network characteristics of three diseases. The 555 CAD-specific DEGs formed the most extensive and complex PPI network, with three key hub genes identified, including CD68, ICAM1, and IL10 (Figures 5C,D). The 44 OSA-specific DEGs generated a relatively simple interaction network yielding eight hub genes, consisting of HBD, ALAS2, NIP1L4, CPA3, HDC, GLUD2, MYZAP, and PKP2 (Figures 5E,F). While the 73 shared CADOSA DEGs produced an intermediate complexity network with six central hub genes, including HP, TIMD4, S100A12, PLBD1, MMP9, and VCAN (Figures 5G,H).

### 3.6 Potential biomarkers of OSA, CAD, and their comorbidity

Through time-series analysis of DEGs, we characterized dynamic gene expression patterns during disease progression from healthy controls (HC) to CAD/OSA and subsequently to CADOSA comorbidity. In the “HC→CAD→CADOSA” transition, we identified four expression clusters (Supplementary Figure S1): an upward-trending cluster (81 genes) peaking in CADOSA, a downward-trending cluster (180 genes), an inverted V-shaped cluster (580 genes) maximal in CAD, and a V-shaped cluster (120 genes). Similar analysis of “HC→OSA→CADOSA” revealed four patterns (Supplementary Figure S2): an upward-trending cluster (260 genes) highest in CADOSA, a V-shaped cluster (73 genes), a downward cluster (84 genes), and an inverted V-shaped cluster (40 genes) peaking in OSA. Integration of these patterns identified 374 CAD-specific core genes (intersecting unique DEGs with CAD-peaking inverted V-shaped patterns), 15 OSA-specific core genes showing similar characteristics, and 8 CADOSA core genes exhibiting upward trends in both progression pathways.

**Figure 6:**
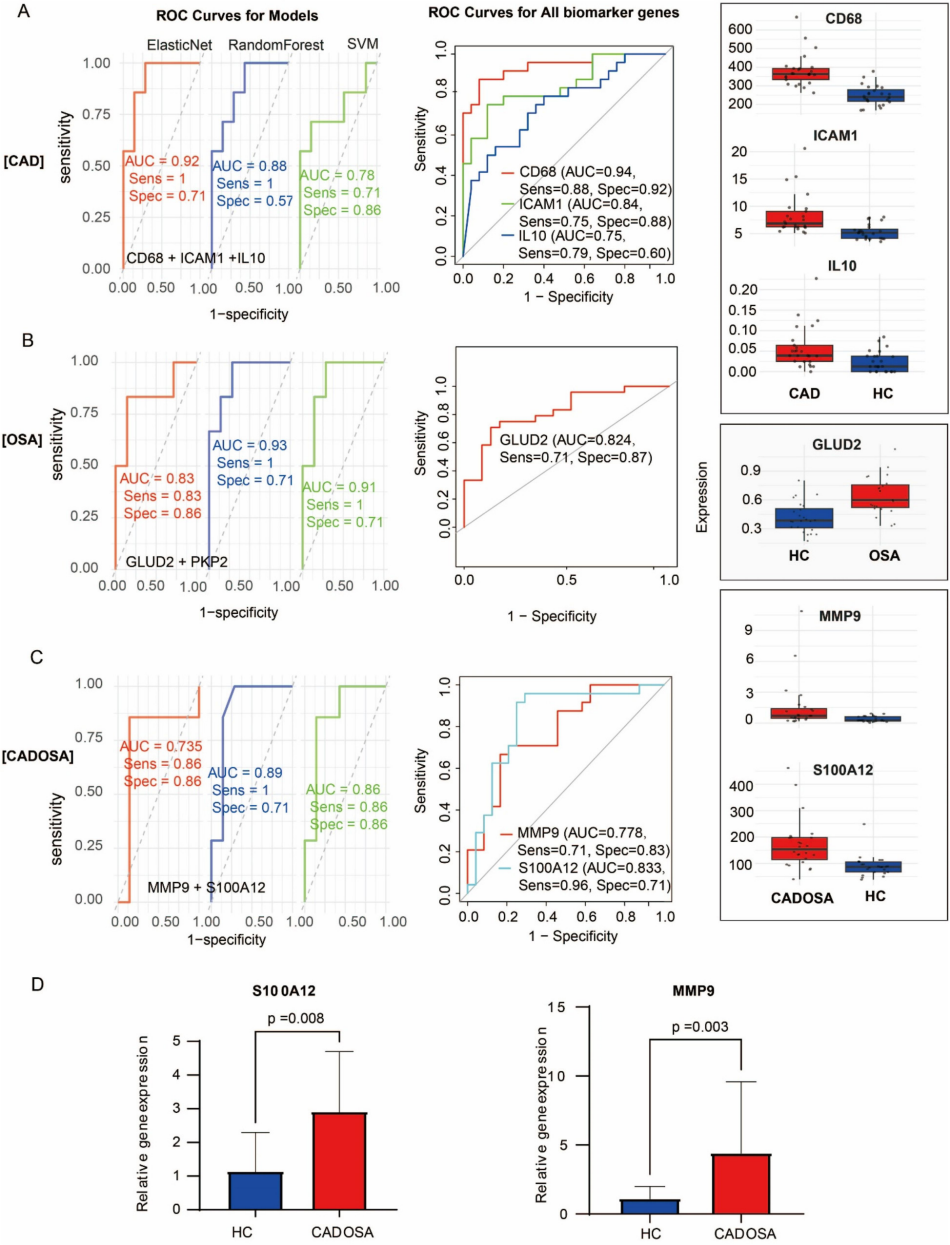
Evaluation of diagnostic models and biomarker performance. **(A)** CAD analysis: (Left) ROC curves comparing the diagnostic performance of Elastic Net, Random Forest, and SVM models; (Middle) ROC analysis of individual biomarkers (CD68, ICAM1, IL10); (Right) Expression levels of the three biomarkers in CAD vs. controls. **(B)** OSA analysis: (Left) ROC curves of three machine learning models; (Middle) Diagnostic performance of GLUD2; (Right) GLUD2 expression in OSA vs. controls. **(C)** CADOSA comorbidity analysis: (Left) Model performance comparison; (Middle) ROC analysis of MMP9 and S100A12; (Right) Expression profiles of both biomarkers. **(D)** Expression of MMP9 and S100A12 between CADOSA and health control quantified using qRT-PCR.

Through integration of core genes with hub genes, we identified candidate biomarkers including three CAD-associated (CD68, ICAM1, IL10), two OSA-associated (GLUD2, PKP2), and two CADOSA-associated (MMP9, S100A12) genes. Following rigorous evaluation using three machine learning approaches, we established the final biomarker panels and developed diagnostic models for each condition. The composite diagnostic model demonstrated excellent discriminatory capacity across all conditions. For CAD, the elasticNet-based model achieved an AUC of 0.92, while individual biomarkers CD68, ICAM1, and IL10 showed robust standalone performance with AUCs of 0.94, 0.84, and 0.75, respectively (Figure 6A). CD68 had the highest expression, while IL10 displayed the lowest expression. Similarly, the random forest-derived OSA model exhibited strong diagnostic accuracy (AUC = 0.93) followed by SVM (AUC = 0.91), with GLUD2 maintaining high predictive value as a single biomarker (AUC = 0.82) (Figure 6B). For CADOSA comorbidity, the random forest model incorporating both biomarkers performed optimally with an AUC of 0.89, while individual analysis revealed significant diagnostic capability for S100A12 (AUC = 0.83) and MMP9 (AUC = 0.78) (Figure 6C), confirming their clinical utility as standalone indicators. The S100A12 showed much higher expression level than the MMP9. Subsequent qRT-PCR validation confirmed their significant upregulation in CADOSA patients (Figure 6D), further supporting their potential clinical utility as independent diagnostic indicators.

### 3.7 Immune landscape and their correlation with biomarker genes

**Figure 7:**
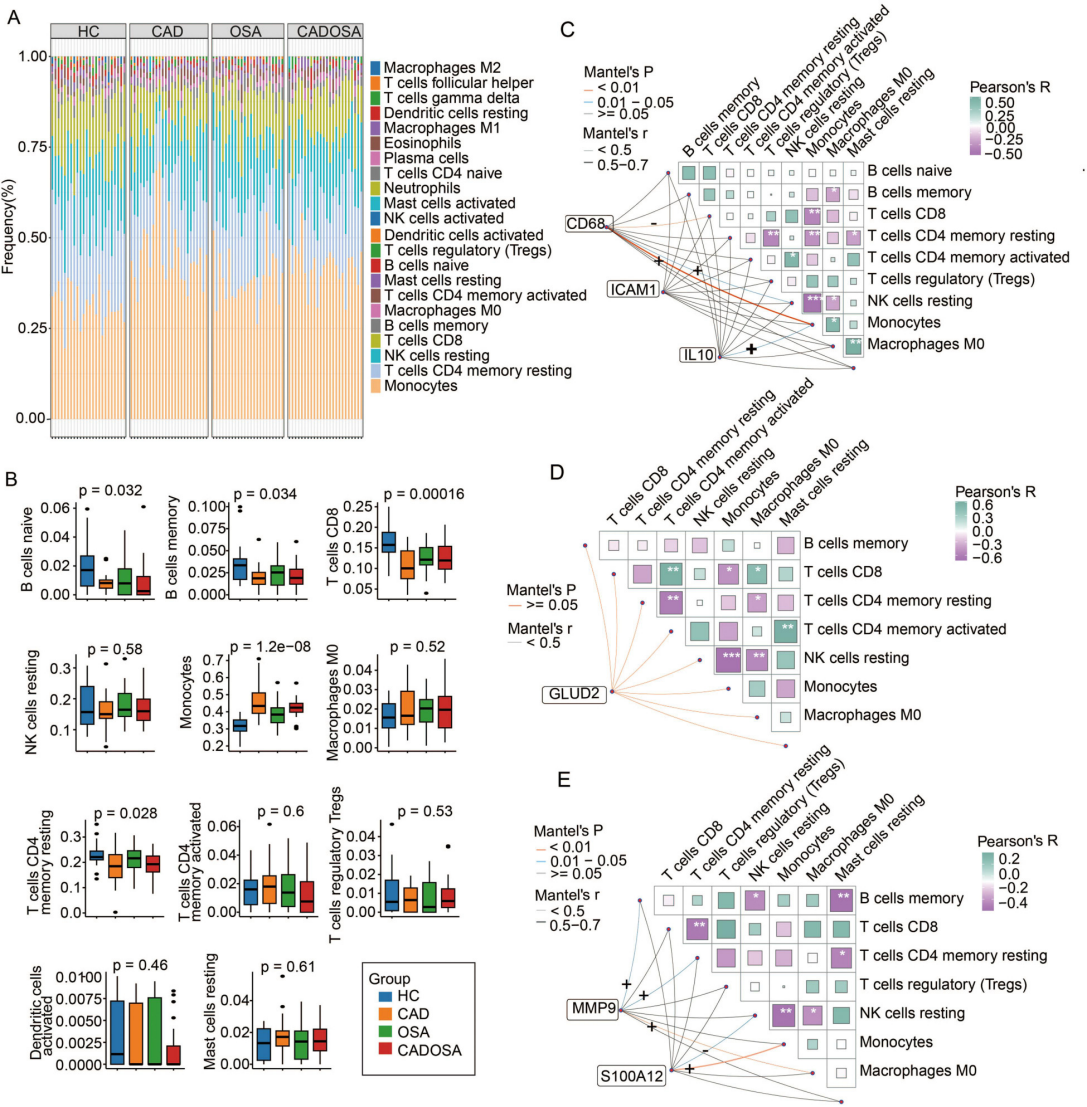
Immune cell profiling and biomarker correlations in healthy controls (HC), CAD, OSA, and CADOSA groups. **(A)** Proportion of 22 cell types estimated by CIBERSORT. **(B)** Differences in the proportion of 11 primary immune cells (detected > 25% samples). **(C)** The correlation between the proportion of immune cells and the expression of biomarker genes CAD68, ICAM1, and IL10 in CAD samples. **(D)** The correlation between the proportion of immune cells and the expression of biomarker genes GLUD2 in OSA samples. **(E)** The correlation between the proportion of immune cells and the expression of biomarker genes MMP9 and S100A12 in CADOSA samples.

Functional enrichment analysis of DEGs highlighted the involvement of immune dysfunctionin the pathogenesis of CAD, OSA, and CADOSA. To further characterize immune dysregulation, we performed immune cell infiltration analysis in PBMCs across the three disease groups. We identified 22 distinct immune cell subtypes, with monocytes being the most abundant, followed by CD4+ memory resting T cells, NK resting cells, and CD8+ T cells, collectively accounting for nearly 80% of PBMCs (Figure 7A). Compared to healthy controls, all three patient groups exhibited a significant reduction in CD4+ memory resting T cells, CD8+ T cells, B memory cells, and B naïve cells, alongside a pronounced increase in monocytes (Figure 7B).

To assess immune cell interactions, we conducted correlation analyses in CAD samples. CD4+ memory resting T cells showed a negative association with regulatory T cells and resting mast cells, while NK resting cells were inversely correlated with macrophages M0 (Figure 7C). Notably, the three most abundant immune subsets—CD4+ memory resting T cells, NK resting cells, and CD8+ T cells—all demonstrated a negative relationship with monocytes. Furthermore, we examined the association between CAD biomarker genes and immune cells. CD68 expression was positively correlated with monocytes and NK resting cells but negatively correlated with CD8+ T cells, whereas IL10 exhibited a positive relationship with monocytes.

In OSA patients, CD4+ memory resting T cells were inversely associated with activated CD4+ memory T cells and macrophages M0. NK resting cells showed a negative correlation with macrophages M0 and monocytes, while CD8+ T cells were positively linked to activated CD4+ memory T cells and macrophages M0 but negatively correlated with monocytes. However, no significant relationship was observed between the OSA biomarker GLUD2 and immune cell proportions (Figure 7D).

For the CADOSA cohort, we found that CD8+ T cell proportions were negatively associated with CD4+ memory resting T cells, and monocytes exhibited an inverse correlation with NK resting cells (Figure 7E). Additionally, we evaluated the relationship between two key CADOSA biomarkers and immune cell infiltration. S100A12 displayed strong positive correlations with monocytes but negative associations with NK resting cells (Figure 7C). Conversely, MMP9 was positively correlated with macrophages M0, CD4+ memory resting T cells, and B memory cells (Figure 7E).

## 4 Discussion

It is common for OSA and CAD to coexist, especially CAD patients frequently present with comorbid OSA, which raises the severity of disease and rates of mortality (20–22). The pathophysiological interaction between OSA and CAD is complex and comprises a variety of elements, including humoral and haemodynamic factors. Uncovering the molecular pathways and key regulators has great significance for both prevention and treatment of this complication. For example, ERBB receptor feedback inhibitor 1 (ERRFI1114) was proposed as a potential biomarker for OSA and atherosclerosis (23). However, the molecular drivers of CADOSA comorbidity are still poorly understood, despite the fact that shared genetic locus genes, such as CDKN2B-AS1, PHACTR1, CELSR2, LPA, CARMAL, MFGE8, and GGT5, have been implicated in both CAD and OSA (24). In order to delineate important regulators and pathways underlying this high-risk multimorbidity and identify its diagnostic molecular biomarkers, we integrated clinical and transcriptomic profiling with comprehensive transcriptomic analyses to fill this gap.

Clinically, the progressive increase in carotid plaque burden, left ventricular hypertrophy, valvular regurgitation, and left ventricular dysfunction (LVD/LVS) from healthy controls (HC) to OSA, CAD, and ultimately CADOSA highlights the escalating cardiovascular dysfunction in comorbid conditions (25). Although some of these clinical factors were not statistically significant, this may be due to the relatively small sample size. Future studies with larger sample sizes should be conducted if possible. Notably, patients with OSA and CADOSA exhibit higher apnea-hypopnea index (AHI) values and lower SpO₂ levels compared to CAD alone, while CADOSA patients further demonstrate elevated triglyceride (TG) and creatinine (CR) levels. These findings suggest that comorbid CADOSA presents more severe clinical manifestations than isolated CAD or OSA, likely due to synergistic hemodynamic stress from atherosclerosis and sleep-disordered breathing. One important observation is the notable sex imbalance across the groups, with a male predominance in the CADOSA cohort (92% male), compared to the healthy control (42% male), OSA (70% male), and CAD (76% male) groups. This disparity may introduce confounding effects in the interpretation of transcriptomic differences, particularly if sex influences gene expression related to cardiovascular or sleep apnea pathways. In addition, other factors such as body size which may necessitate the indexing of echocardiographic measurements to body surface area, comorbidities including hypertension, hyperlipidemia, and hyperglycemia, as well as medication use, could further contribute to variability in gene expression. Future studies with sex-balanced cohorts and careful consideration of these potential confounders are warranted to validate these findings.

At the molecular level, dysfunctional efferocytosis, which is a critical process mediated by phagocytes for clearing apoptotic cells and resolving inflammation, has been implicated in atherosclerosis (26). The highest plaque burden in CADOSA, followed by CAD, aligns with the observed dysregulation of phagosome and efferocytosis pathways. Additionally, neutrophil extracellular traps (NETs), while essential for innate immunity, have recently been recognized as contributors to CAD progression. Our results corroborate this, showing significant activation of NET formation pathways in both CAD and CADOSA. Furthermore, cytoskeletal abnormalities, such as desmin and dystrophin dysfunction, are known to impair pharyngeal muscle function in OSA (27). The enrichment of the “cytoskeleton in muscle cells” pathway in OSA and CADOSA underscores the systemic impact of cytoskeletal dysregulation beyond localized tissue effects.

Except the shared pathways, distinct cellular processes were characterized for each disease condition. In CAD, immune activation is evident through the enrichment of hematopoietic cell lineage, complement and coagulation cascades, and B-cell receptor signaling pathways. Lipid metabolism dysregulation, particularly in ether lipid and arachidonic acid metabolism, further supports the well-documented metabolic imbalance in CAD (28–30). Additionally, perturbations in amino acid metabolism, such as tryptophan depletion which exacerbates ischemic vascular disease by impairing protein and hemoglobin synthesis, highlight the multifaceted metabolic dysfunction in CAD (31). Blood histamine has been identified as a risk factor for coronary events (32), Histamine is produced through the decarboxylation of histidine, and the observed enrichment of histidine metabolism suggests its potential contribution to CAD pathogenesis. These findings indicate that CAD may represent a systemic immune-metabolic dysfunction. In OSA, the inhibition of the PI3K-Akt signaling pathway has been proposed as a therapeutic target (33). while alterations in nitrogen metabolism, consistent with prior findings in urinary metabolites (34), suggest systemic metabolic disturbances. The enrichment of cholinergic synapse pathways may reflect impaired upper airway neuromuscular control (34), though its precise role in OSA pathogenesis warrants further investigation. Notably, the association between arrhythmogenic right ventricular cardiomyopathy pathways and OSA provides a molecular basis for the elevated CAD risk in OSA patients. For CADOSA, the predominant enrichment of immune related NOD-like receptor and metabolism associated PPAR signaling pathways points to a unique immune-metabolic imbalance distinct from CAD alone. These pathways, which integrate inflammatory and metabolic regulation (35–37), may underlie the exacerbated pathophysiology observed in CADOSA patients.

This study systematically identified potential biomarkers for CAD-OSA comorbidity through an integrative bioinformatics approach. However, the relatively small sample size may limit statistical power, and validation in an independent cohort is needed in future studies. To enhance reliability and mitigate overfitting, we employed multiple strategies, including rigorous bioinformatic dimensionality reduction, 10-fold cross-validation, and experimental validation by qPCR. By combining stepwise DEG analysis, expression pattern-based core gene selection, PPI network-derived hub gene classification, and machine learning validation, we established a robust framework for biomarker discovery. The core hub gene-based predictive models demonstrated strong diagnostic performance across disease conditions, with ElasticNet, Random Forest, and SVM algorithms each showing optimal efficacy for specific disease states. Notably, single biomarkers exhibited high predictive value (as evidenced by AUC metrics), though their combined use only marginally improved diagnostic accuracy, supporting their utility as independent indicators. Among the identified biomarkers, CD68 emerged as a particularly promising candidate for CAD, showing both the highest AUC and expression levels. Its strong correlation with monocyte infiltration aligns with its established role as a macrophage/monocyte marker in inflammatory processes (38). Our findings further corroborate previous reports implicating ICAM1 and IL-10 in cardiovascular pathogenesis (39–41), validating the reliability of our integrative methodology. For OSA, we identified GLUD2 as a novel biomarker encoding the hGDH2 isoform of glutamate dehydrogenase (41). This enzyme plays a central role in glutamate metabolism, a key neurotransmitter pathway implicated in neurological aspects of OSA pathogenesis (42, 43). While these findings suggest a plausible mechanistic link, the precise role of GLUD2 in OSA development warrants further investigation. S100A12, a calgranulin family protein involved in inflammatory regulation, has been proposed as a biomarker for both CAD (44) and OSA (45). Its positive correlation with monocyte levels suggests that it acts as a key mediator of monocyte-driven inflammation, thereby amplifying vascular dysfunction and atherosclerosis the context of comorbid OSA and CAD. MMP9 (matrix metalloproteinase-9) is a protease that degrades and remodels the extracellular matrix (ECM), and its elevation exacerbates inflammatory cell migration and plaque instability/rupture which probably contribute to comorbidity of CAD and OSA (46). MMP9 has been linked to CAD and OSA morbidity (47), with further evidence supporting its utility as an OSA biomarker (48). Their consistent association with disease morbidity and strong diagnostic performance with qPCR validation highlights their potential as cross-condition biomarkers.

Our study delineates both shared and distinct molecular pathways underlying OSA, CAD, and their comorbidity, while proposing new therapeutic targets. The discovered biomarkers could potentially be developed into a clinical assay to aid in risk stratification and management. Furthermore, these biomarkers could serve as objective endpoints in clinical trials, testing the efficacy of therapies aimed at mitigating the shared pathophysiology of this comorbidity. Although future validation is needed, our study provides a crucial foundation for personalized management in this comorbid population. Nevertheless, certain limitations must be acknowledged. Firstly, our PBMC-based profiling offers valuable insights, it may not fully reflect tissue-specific responses, particularly within vascular endothelium or atherosclerotic plaques, which is warrant further investigation. Secondly, the mechanistic contributions of identified biomarkers to CADOSA pathogenesis requires further mechanic investigation through *in vivo* and *in vitro* experiments, which is essential for the development of targeted therapies for CADOSA comorbidity.

## 5 Conclusion

Patients with CADOSA comorbidity demonstrate significantly exacerbated clinical manifestations compared to isolated CAD or OSA, including more severe cardiovascular dysfunction, pronounced sleep-disordered breathing, and aggravated metabolic disturbances. Our study elucidates the molecular mechanisms underlying this high-risk multimorbidity, revealing both shared pathological pathways (including impaired efferocytosis, neutrophil extracellular trap activation, and cytoskeletal abnormalities) and distinct molecular signatures (particularly NOD-like receptor and PPAR signaling pathways). We identified GLUD2, S100A12, and MMP9 as robust diagnostic biomarkers, with GLUD2 reflecting glutamate metabolism-related neural dysfunction in OSA and S100A12/MMP9 indicating cross-condition inflammatory processes in CADOSA. These findings not only provide a comprehensive molecular framework explaining the heightened severity of CADOSA but also offer clinically valuable biomarkers for early detection and targeted therapeutic intervention, advancing toward precision medicine approaches for this complex comorbidity.

## Data Availability

The datasets presented in this study can be found in online repositories. The names of the repository/repositories and accession number(s) can be found below: https://www.ncbi.nlm.nih.gov/, PRJNA1248123.
