# Supplementary figures and images for: Integrative transcriptomic analysis reveals diagnostic biomarkers for comorbidity of coronary artery disease and obstructive sleep apnea
Source: Front Cardiovasc Med. 2025 Sep 17;12:1658016. doi: 10.3389/fcvm.2025.1658016 (PMC12484168; doi:10.3389/fcvm.2025.1658016)

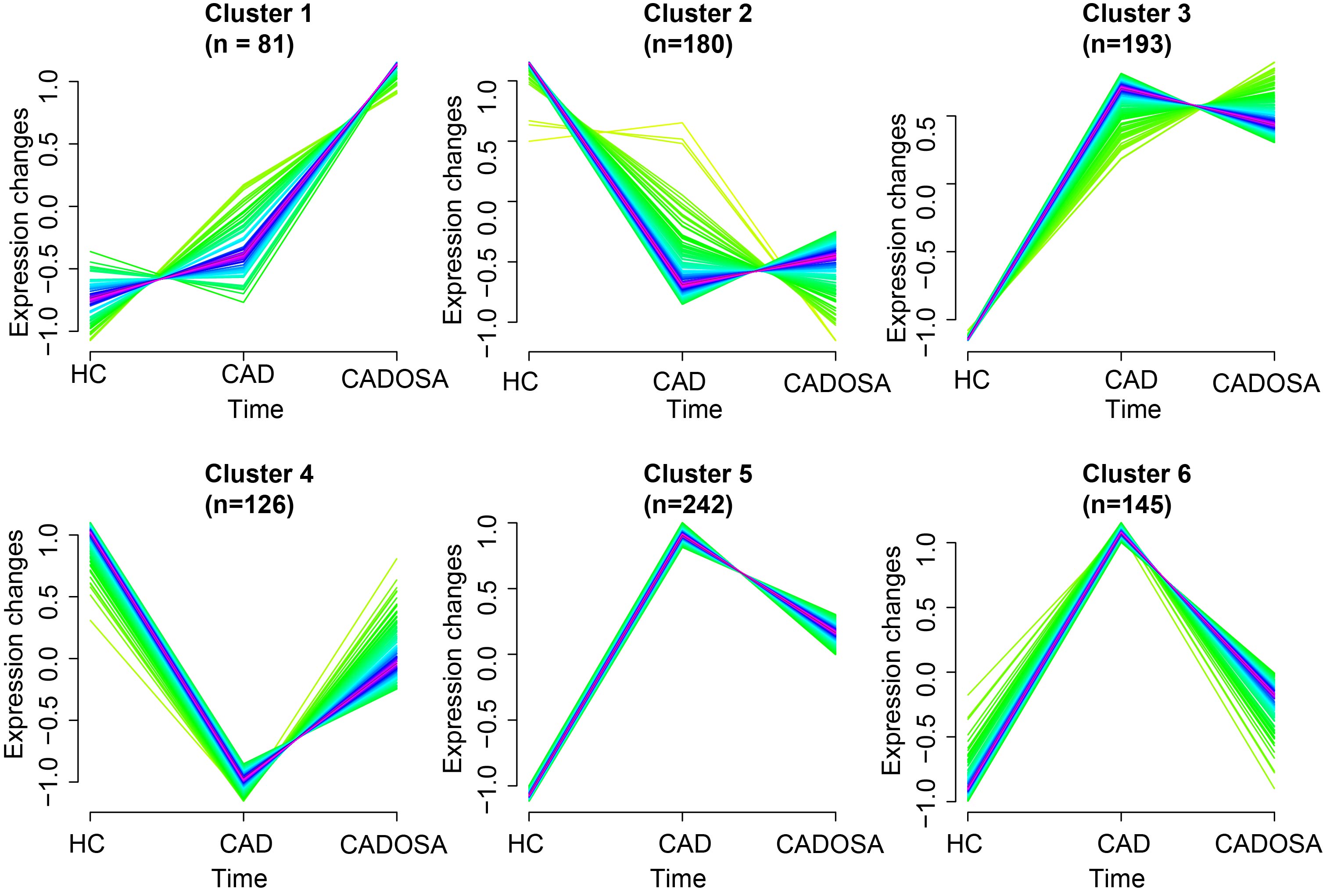

Supplement: Supplementary file 1 [file Datasheet1.zip › FigS1.jpg]

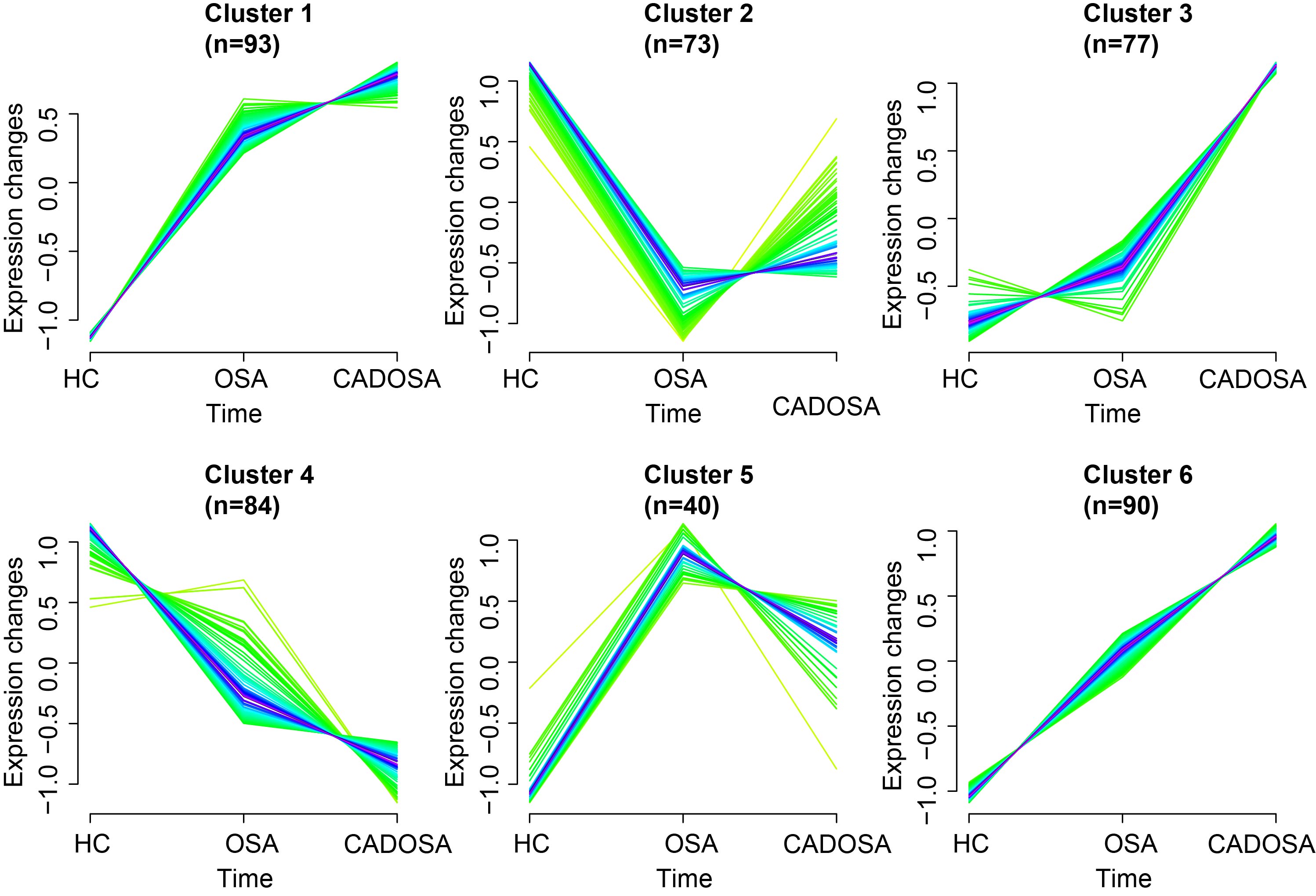

Supplement: Supplementary file 1 [file Datasheet1.zip › FigS2.jpg]
